# Analysis of chronic kidney disease among national hospitalization data with 14 million children

**DOI:** 10.1186/s12882-021-02383-1

**Published:** 2021-05-25

**Authors:** Xinmiao Shi, Ying Shi, Luxia Zhang, Lanxia Gan, Xuhui Zhong, Yuming Huang, Chen Yao, Yanfang Wang, Chongya Dong, Beini Liu, Fang Wang, Haibo Wang, Jie Ding

**Affiliations:** 1 Department of Pediatrics, Peking University First Hospital, Beijing, China; 2 China Standard Medical Information Research Center, Shenzhen, China; 3 Renal Division, Department of Medicine, Peking University First Hospital, Beijing, China; 4 National Institute of Health Data Science at Peking University, Beijing, China; 5 Department of Biostatistics, Peking University First Hospital, Beijing, China; 6 Peking University Clinical Research Institute, Beijing, China; 7 Clinical Trial Unit, First Affiliated Hospital of Sun Yat-Sen University, Guangzhou, China

**Keywords:** Chronic kidney disease, Child, Hospitalization, Database

## Abstract

**Background:**

The main purpose was to determine basic epidemiological data on CKD among hospitalized pediatric patients in China.

**Methods:**

Data from pediatric inpatients with CKD hospitalized from June 1, 2013 to May 31, 2017 were extracted from the electronic records of HQMS database, which includes over 14 million inpatients. Codes from the 10th revision of the International Classification of Diseases (ICD-10) were used to search the database.

**Results:**

A total of 524 primary diseases of CKD were included in this study. In all, there were 278 231 pediatric inpatients with CKD, which accounted for 1.95 % of the 14 250 594 pediatric inpatients registered in the HQMS database. The number of pediatric inpatients with CKD was 67 498 in 2013, 76 810 in 2014, 81 665 in 2015 and 82 649 in 2016, which accounted for 1.93 %, 1.93 %, 1.99 and 2.09 %, respectively, of the total population of pediatric inpatients. The etiology of CKD was secondary nephrosis in 37.95 % of cases, which ranked first and followed by CAKUT with a percentage of 24.61 %. Glomerular diseases and cystic kidney disease accounted for 21.18 and 5.07 %, respectively. Among all 278 231 patients, 6 581 (2.37 %) had a primary discharge diagnosis of CKD. The renal pathology findings of CKD showed that IgA accounted for 51.17 %.

**Conclusions:**

This study provides a descriptive analysis of the hospitalized population of pediatric CKD patients. Our study provides important, fundamental data for policy making and legislation, registry implementation and the diagnosis, treatment and prevention of CKD in China.

**Supplementary Information:**

The online version contains supplementary material available at 10.1186/s12882-021-02383-1.

## Background

Chronic kidney disease (CKD) is defined as an abnormality of kidney structure or function confirmed with pathological, laboratory or imaging findings, or a GFR < 60 ml/min/1.73 m^2^, present for more than 3 months, with implications for health [1]. CKD has become a major public health concern worldwide and involves biological, social and psychological burdens for patients [2, 3]. The progression of CKD to end-stage renal disease (ESRD) is associated with high mortality and cardiovascular morbidity [4]. Therefore, prevention, diagnosis and treatment of CKD are of vital importance.

Early detection of CKD is essential for preventing progression to ESRD, which is in need of the epidemiological data of CKD [4]. Numerous studies have been conducted in adult populations worldwide and epidemiological data are available. The USA [5] and Norway [6] have reported CKD prevalence of 13.0 and 10.2 %, respectively. A national survey in China reported an overall CKD prevalence of 10.8 % among people aged 18 years and older [7]. The epidemiology of childhood ESRD has been analyzed in the USA, Europe, Australia and New Zealand [8]; however, these data represent only part of the pediatric population with CKD, because many children with renal impairment will not develop ESRD until adulthood [9]. There is a paucity of data on the epidemiology of CKD in children worldwide, especially in developing countries like China [6]. To date, no nationwide epidemiological data on childhood CKD have been reported; the only nationwide study was conducted by the Chinese Society of Pediatric Nephrology and focused on chronic renal failure (CRF), not CKD, in 91 hospitals in China in 2004 [10]. That study was performed over 14 years ago and included only a very small proportion of hospitals. Moreover, patients who do not meet the definition of CRF can also potentially progress to ESRD. As reported, over 9 % of patients with stage 3 or 4 CKD progress to ESRD [11].

It is time-consuming, labor-intensive and dear to conduct population-based researches in China with such a big population of over 1.3 billion. Therefore, we aimed to use the national database of the Hospital Quality Monitoring System (HQMS) to explore the epidemiology of childhood CKD by evaluating patients hospitalized in China between June 1, 2013 and May 31, 2017.

## Methods

### Study design

This study is a descriptive analysis of pediatric CKD patients aged less than 18 years who were hospitalized from June 1, 2013 through May 31, 2017. Data were obtained from the HQMS database. All methods were carried out in accordance with relevant guidelines and regulations.

The renal diseases of childhood CKD were determined according to *Pediatric Nephrology* (7th version) [12], *Zhufutang’s Practical Pediatrics* (8th version) [13] and *Nephrology* (3rd version) [14]. Patients with at least one of the following discharge diagnoses as either a primary or secondary diagnosis were identified as having CKD: glomerular diseases, including hereditary glomerular diseases, congenital glomerular diseases, and primary glomerular diseases; tubular diseases and tubular interstitial diseases, including hereditary/congenital tubular diseases, primary tubular disease, and tubular interstitial diseases; systemic diseases, including Henoch-Schonlein purpura nephritis (HSPN), lupus nephritis (LN), hemolytic uremic syndrome (HUS), renal injury related to autoimmune diseases or connective tissue diseases, and others (renal injury related to metabolic disorders, renal injury related to infectious disorders, renal injury related to drugs, renal vascular thrombosis or embolism, thrombotic microangiopathy, antiphospholipid syndrome, and hypertensive renal damage); congenital anomalies of kidney and urinary tract (CAKUT; renal hypoplasia, dysplasia, oligonephronia, obstructive uropathies and reflux nephropathy, and cystic kidney diseases); renal injury related to tumors; CRF of unknown cause; and others. The following diseases were excluded: acute kidney injury, symptoms and signs related to the urinary system, syndromic kidney damage, urinary system lithiasis, urinary system infection, and renal diseases without exact codes in the 10th revision of the International Classification of Diseases (ICD-10).

In China, use of the International Classification of Diseases was mandated by the National Health Commission of the People’s Republic of China. However, many coding variations were generated during the development of hospital information systems [15]. Therefore, three editions of the ICD-10 coding system were utilized for data capture in this study: the China edition, Beijing edition and Clinical edition. These three editions of ICD-10 codes for every renal disease of CKD were used for data capture.

### Data source

Data were captured from the HQMS database. The HQMS is a mandatory national database of hospitalized patients that is managed by the National Health Commission of the People’s Republic of China [16]. Since January 1, 2013, every tertiary hospital in China has been required to automatically submit electronic medical records to the HQMS daily. Automatic data quality control for completeness, consistency, accuracy and safety is guaranteed at the time of data uploading. The data achieved stability beginning in June 2013 [17].

The database includes data collected from 993 tertiary hospitals in 31 provinces throughout China between June 1, 2013 and May 31, 2017.

For each patient in the HQMS database, electronic medical records include demographic characteristics, clinical diagnoses, procedures, pathology diagnoses and expenditures. Informed consent was obtained from guardians of inpatient children on admission to hospital.

The discharge diagnoses are coded with ICD-10 codes by certified professional medical coders at every hospital.

### Data capture and analysis

Cases of CKD were captured from the database by using codes for the primary diseases of CKD from three editions of the ICD-10.

Although patients could not be identified by their identification numbers directly because of de-identification and data encryption in the HQMS database, the same patients hospitalized at different hospitals could be identified by their demographic information. The rate of identification exceeds 91 % when name, sex and date of birth are used concurrently.

The study period was divided into four groups: June 2013 through May 2014, June 2014 through May 2015, June 2015 through May 2016 and June 2016 through May 2017. These date ranges were labeled “2013,” “2014,” “2015” and “2016” in this study.

**Fig. 1:**
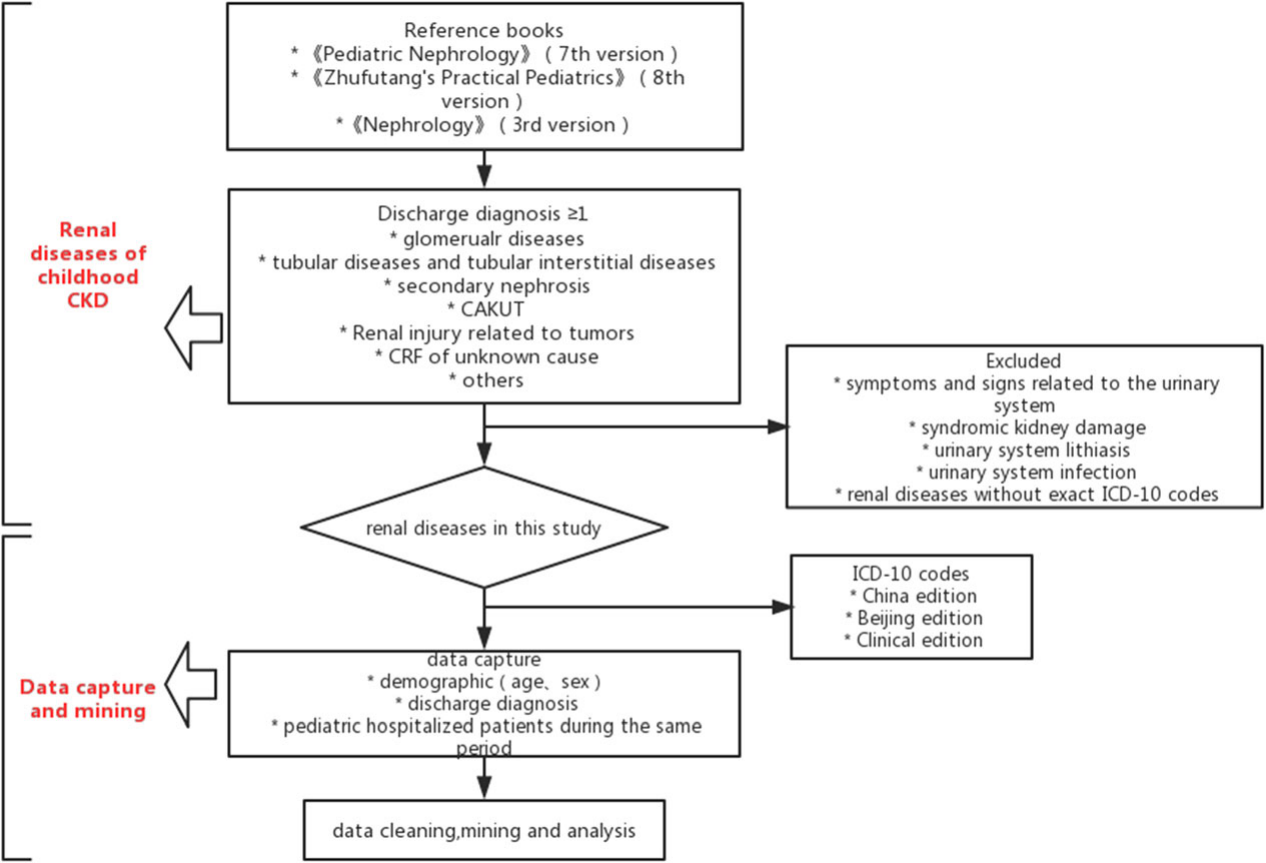
Flowchart of study

If a patient had multiple diagnoses of primary diseases of CKD during hospitalization, all diagnoses were retained in the analysis of etiology. The flowchart of this study was shown in Fig. 1.

### Statistical analysis

Continuous data were described as mean with standard deviation; categorical variables were presented as frequency and proportions. A descriptive analysis was performed to determine the basic epidemiological data of CKD in children in China. All statistical analyses were performed with SAS software (version 9.4, North Carolina) and R (version 3.5.1, Lucent Technologies).

## Results

The three editions of ICD-10 codes for renal diseases of CKD are listed in Additional File 1. The total number of pediatric inpatients with CKD in the study period was 278 231, which accounted for 1.95 % of the 14 250 594 pediatric inpatients in the HQMS from 2013 to 2016. The male/female ratio was 1.80:1 (Table 1).

**Table 1:** Demographic features of CKD patients in childhood population

	No. of cases	Percent (%)
Number	278,231	
Male (%)	178,561	64.18 %
Female (%)	98,964	35.57 %
Unknown (%)	706	0.25 %
Mean age at the time of diagnosis	6.70 ± 5.78 years	
Age distribution (years)		
0–1	60,116	21.61 %
1–3	34,796	12.51 %
3–6	41,036	14.75 %
6–12	69,545	25 %
12–18	72,738	26.14 %
CKD stage^a^		
CKD stage 1	1611	21.65 %
CKD stage 2	955	12.83 %
CKD stage 3	1361	18.29 %
CKD stage 4	856	11.50 %
CKD stage 5	2659	35.73 %

^a^CKD was the discharge diagnosis in 6581 patients, which accounted for 2.37 % of the 278,231 patients with renal disease

**Fig. 2:**
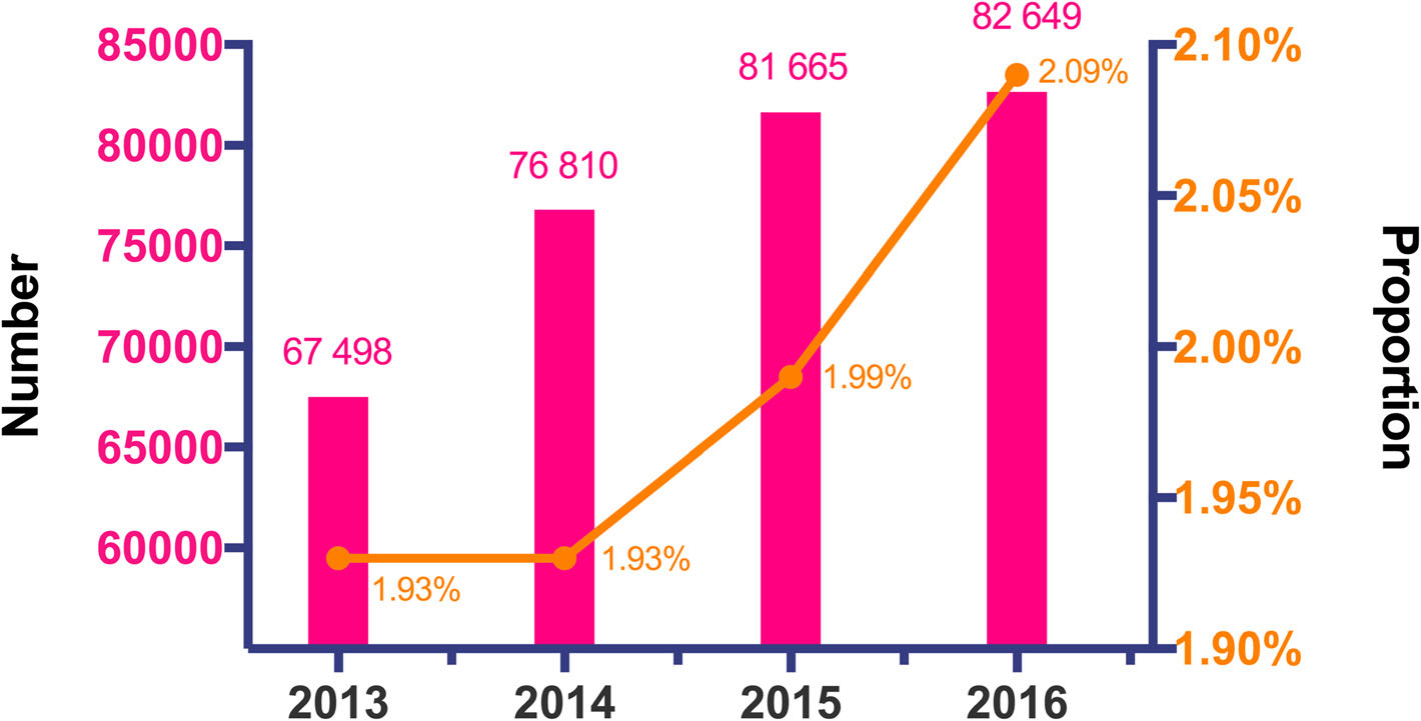
Number and proportion of pediatric CKD patients among all childhood inpatients

The number of pediatric inpatients with CKD was 67 498 in 2013, 76 810 in 2014, 81 665 in 2015 and 82 649 in 2016. The corresponding percentages of pediatric inpatients with CKD among all pediatric inpatients during the same years were 1.93 %, 1.93 %, 1.99 and 2.09 %, respectively (Fig. 2).

**Fig. 3:**
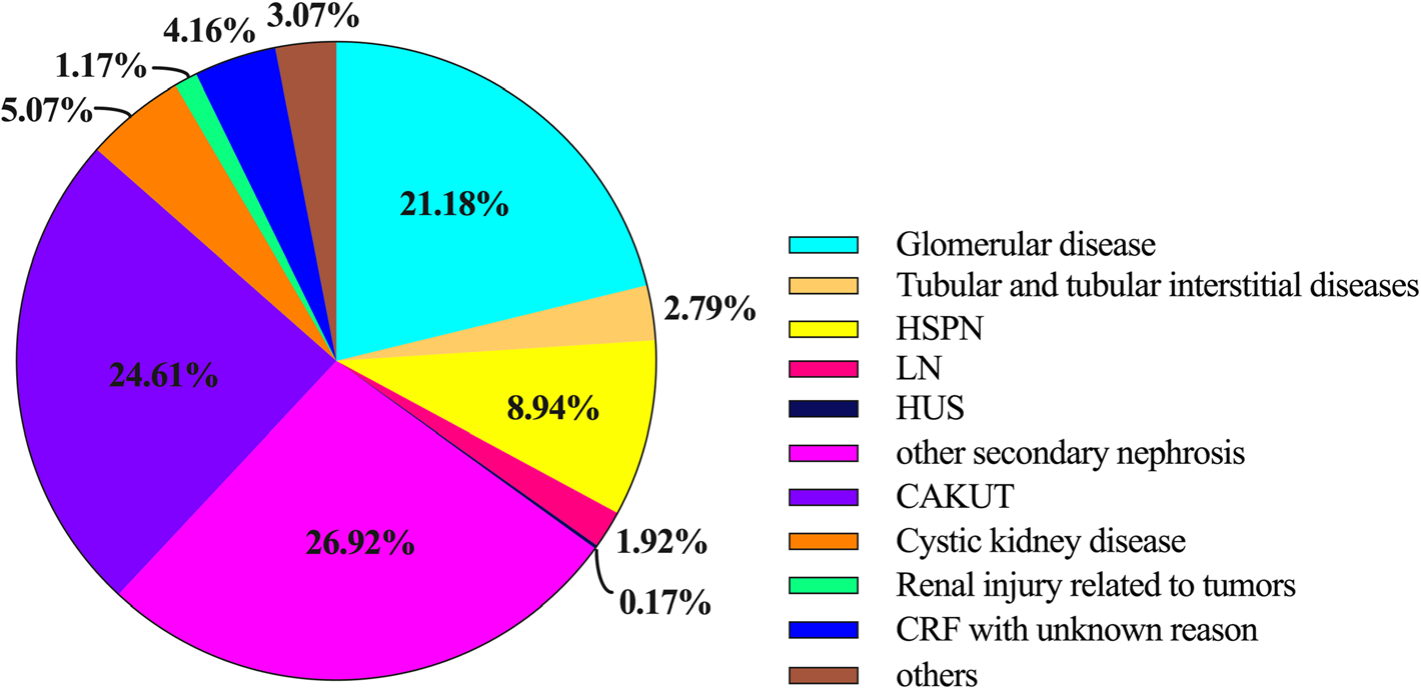
Etiology of CKD

The etiology of CKD was secondary nephrosis in 37.95 % of cases, in which the percentages of HSPN, LN and HUS cases were 8.94 %, 1.92 and 0.17 %, respectively. The percentage of secondary nephrosis ranked first and followed by CAKUT with a percentage of 24.61 %. Glomerular diseases and cystic kidney disease accounted for 21.18 and 5.07 %, respectively. Tubular disease and tubular interstitial diseases accounted for 2.79 % of cases, including hereditary/congenital tubular disease, primary tubular disease and tubular interstitial diseases, which accounted for 1.08 %, 0.06 and 1.65 % of cases, respectively (Fig. 3). The proportion of CAKUT cases showed an increasing trend each year, increasing steadily from 21.68 % to 2013 to 24.68 % in 2016. The proportion of glomerular diseases gradually decreasing from 24.89 % to 2013 to 20.93 % in 2016 (Table 2).

**Table 2:** Etiology of CKD in childhood population during 2013~2016

	No. of patients (%)				
	Total	2013	2014	2015	2016
Glomerular disease	73,806 (21.18 %)	21,077 (24.89 %)	22,195(23.31 %)	23,117(23.00 %)	21,469(20.93 %)
Hereditary glomerular disease	2502 (0.72 %)	615 (0.73 %)	619(0.65 %)	825(0.82 %)	845(0.82 %)
Congenital glomerular disease	418 (0.12 %)	96 (0.11 %)	111(0.12 %)	127(0.13 %)	106(0.10 %)
Primary glomerular disease	70,886 (20.34 %)	20,366 (24.05 %)	21,465(22.54 %)	22,165(22.05 %)	20,518(20.01 %)
Tubular and tubular interstitial diseases	9717 (2.79 %)	2376 (2.81 %)	2595(2.73 %)	2747(2.74 %)	2764(2.70 %)
Hereditary/congenital renal tubular disease	3758 (1.08 %)	979 (1.16 %)	1047(1.1 %)	1045(1.04 %)	1119(1.09 %)
Primary renal tubular disease	224 (0.06 %)	50 (0.06 %)	63(0.07 %)	67(0.07 %)	48(0.05 %)
Renal tubular interstitial disease	5735 (1.65 %)	1347 (1.59 %)	1485(1.56 %)	1635(1.63 %)	1597(1.56 %)
Secondary nephrosis	132,305 (37.95 %)	31,765 (37.50 %)	35,758(37.56 %)	37,265(37.09 %)	39,482(43.94 %)
HSPN	31,167 (8.94 %)	8278 (9.77 %)	8438(8.86 %)	8006(7.97 %)	8606(8.39 %)
LN	6701 (1.92 %)	2050 (2.42 %)	2229(2.34 %)	2342(2.33 %)	2353(2.29 %)
HUS	593 (0.17 %)	158 (0.19 %)	149(0.16 %)	183(0.18 %)	174(0.17 %)
Other nephrosis	93,844 (26.92 %)	21,279 (25.13 %)	24,942 (26.19 %)	26,734 (26.6 %)	28,349 (27.65 %)
Renal injury related to autoimmune and connective tissue diseases	56,346 (16.14 %)	14,145 (16.70 %)	15,684 (16.47 %)	15,821 (15.74 %)	16,199 (15.80 %)
Renal injury related to metabolic disorders	33,064 (9.49 %)	6061 (7.16 %)	8022(8.43 %)	9634(9.59 %)	11,000(10.73 %)
	No. of patients (%)				
	合计	2013	2014	2015	2016
Renal injury related to infectious disorders	1212 (0.35 %)	314 (0.37 %)	337(0.35 %)	319(0.32 %)	289(0.28 %)
Renal injury related to drugs	196 (0.06 %)	49 (0.06 %)	49(0.05 %)	56(0.06 %)	43(0.04 %)
Renal vascular thrombosis and embolism	233 (0.07 %)	79 (0.09 %)	56(0.06 %)	50(0.05 %)	49(0.05 %)
Thrombotic microangiopathy	197 (0.06 %)	145 (0.17 %)	22(0.02 %)	11(0.01 %)	20(0.02 %)
Antiphospholipid syndrome	128 (0.04 %)	41 (0.05 %)	25(0.03 %)	34(0.03 %)	34(0.03 %)
Hypertensive renal damage	2468 (0.71 %)	445 (0.53 %)	747(0.78 %)	809(0.80 %)	715(0.70 %)
CAKUT	85,745 (24.61 %)	18,360 (21.68 %)	22,297(23.41 %)	23,974(23.85 %)	25,310(24.68 %)
Renal hypoplasia, dysplasia, oligonephronia	37,137 (10.66 %)	7547 (8.91 %)	9239(9.7 %)	10,013(9.96 %)	11,640(11.35 %)
Obstructive and recurrent urinary tract disease	48,608 (13.95 %)	10,813 (12.77 %)	13,058(13.71 %)	13,960(13.89 %)	13,670(13.33 %)
Cystic kidney disease	17,668 (5.07 %)	3811 (4.50 %)	4584(4.81 %)	5329(5.3 %)	5339(5.21 %)
Renal injury related to tumors	4091 (1.17 %)	1120 (1.32 %)	1218(1.28 %)	1321(1.31 %)	1269(1.24 %)
CRF with unknown reason	14,485 (4.16 %)	3780 (4.46 %)	3771(3.96 %)	3864(3.84 %)	4120(4.02 %)
Others	10,699 (3.07)	2404 (2.84 %)	2796(2.94 %)	2883(2.87 %)	2787(2.72 %)

**Fig. 4:**
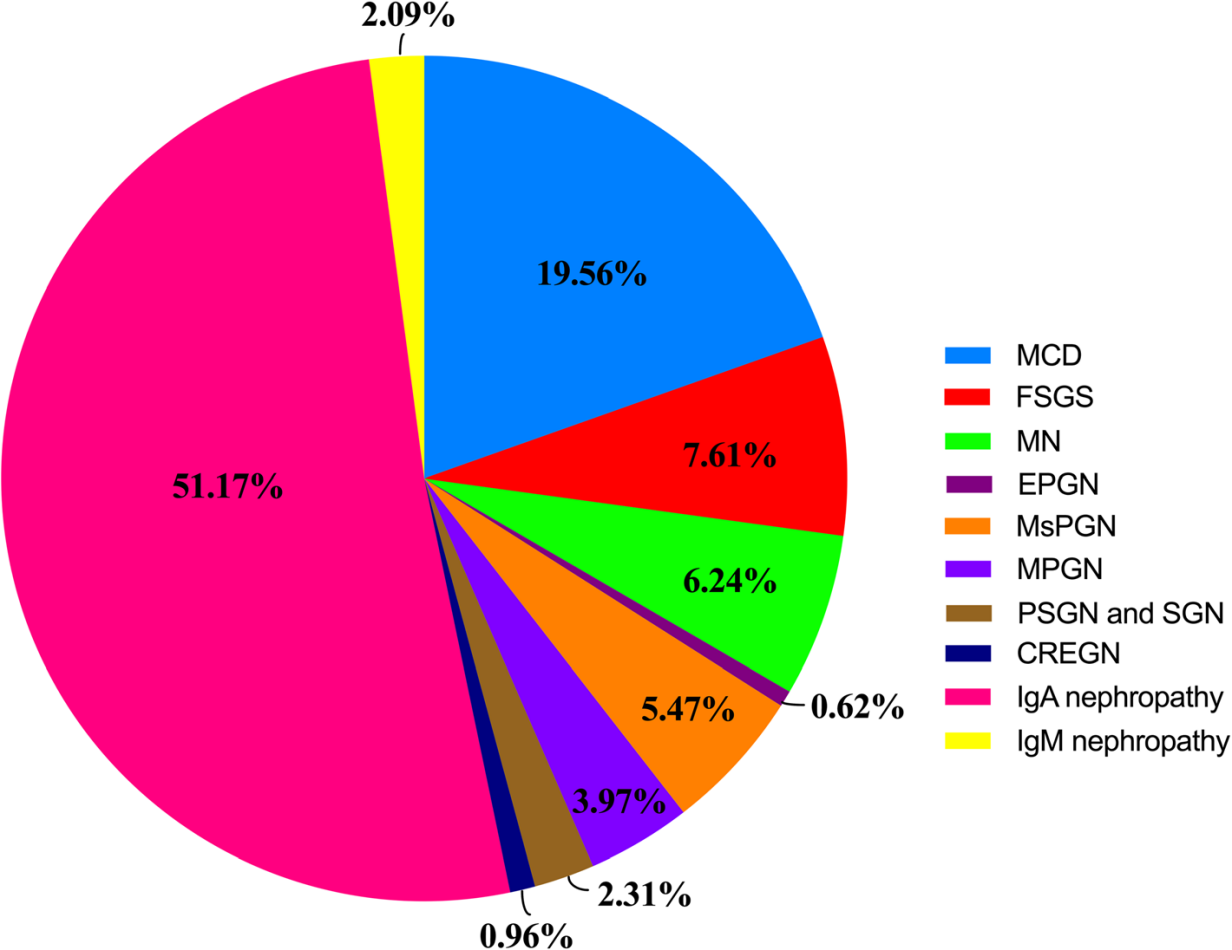
Renal biopsy frequencies of CKD

A total number of 17 418 patients had outcomes of renal biopsies. The renal biopsy frequencies of CKD showed that IgA accounted for 51.17 % and followed by minimal change diseases with a proportion of 19.56 %. The frequencies of other renal biopsies were shown in Fig. 4.

**Fig. 5:**
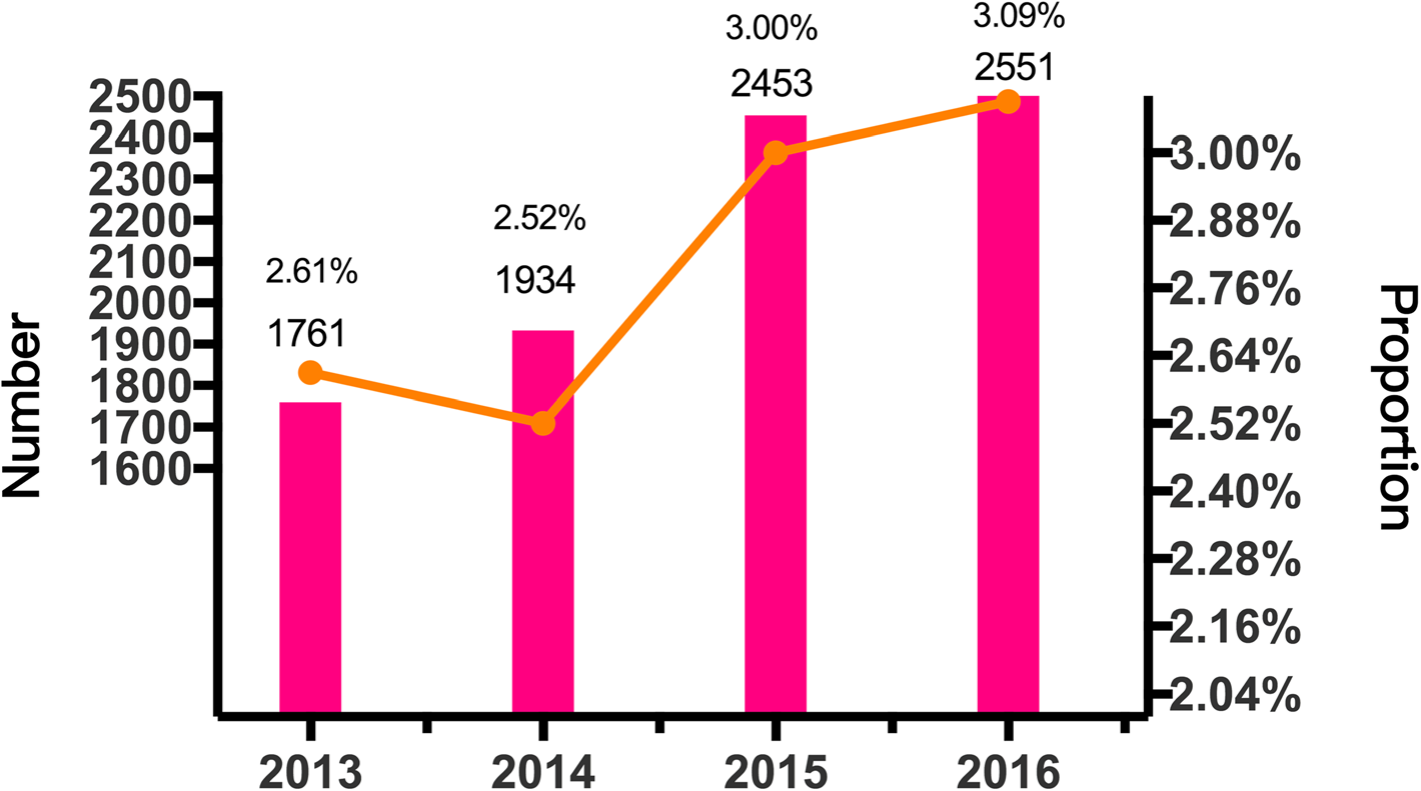
Number and proportion of pediatric CKD-label patients

CKD was the discharge diagnosis (“CKD-label”) in 6581 patients, which accounted for 2.37 % of the 278,231 patients with renal disease. There were 1761 CKD-label patients in 2013, 1934 in 2014, 2453 in 2015 and 2551 in 2016; these accounted for 2.61 %, 2.52 %, 3.00 and 3.09 %, respectively, of the total number of pediatric CKD patients (Fig. 5).

**Fig. 6:**
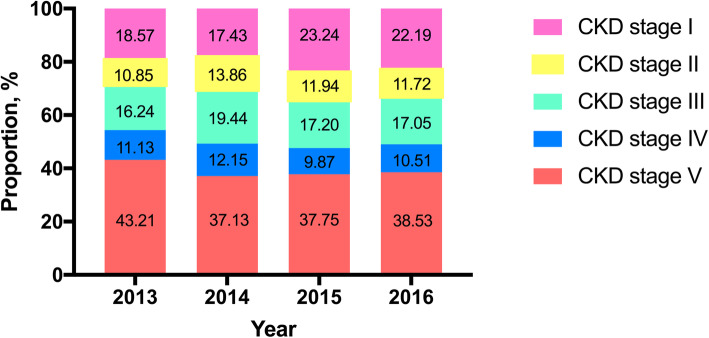
Proportion of each CKD stage among CKD-label patients

Among CKD-label patients, 21.65 % were CKD stage 1, 12.83 % were stage 2, 18.29 % were stage 3, 11.50 % were stage 4 and 35.73 % were stage 5. In each year, a higher proportion of patients were in CKD stage 5 than in any other CKD stage (Fig. 6).

**Fig. 7:**
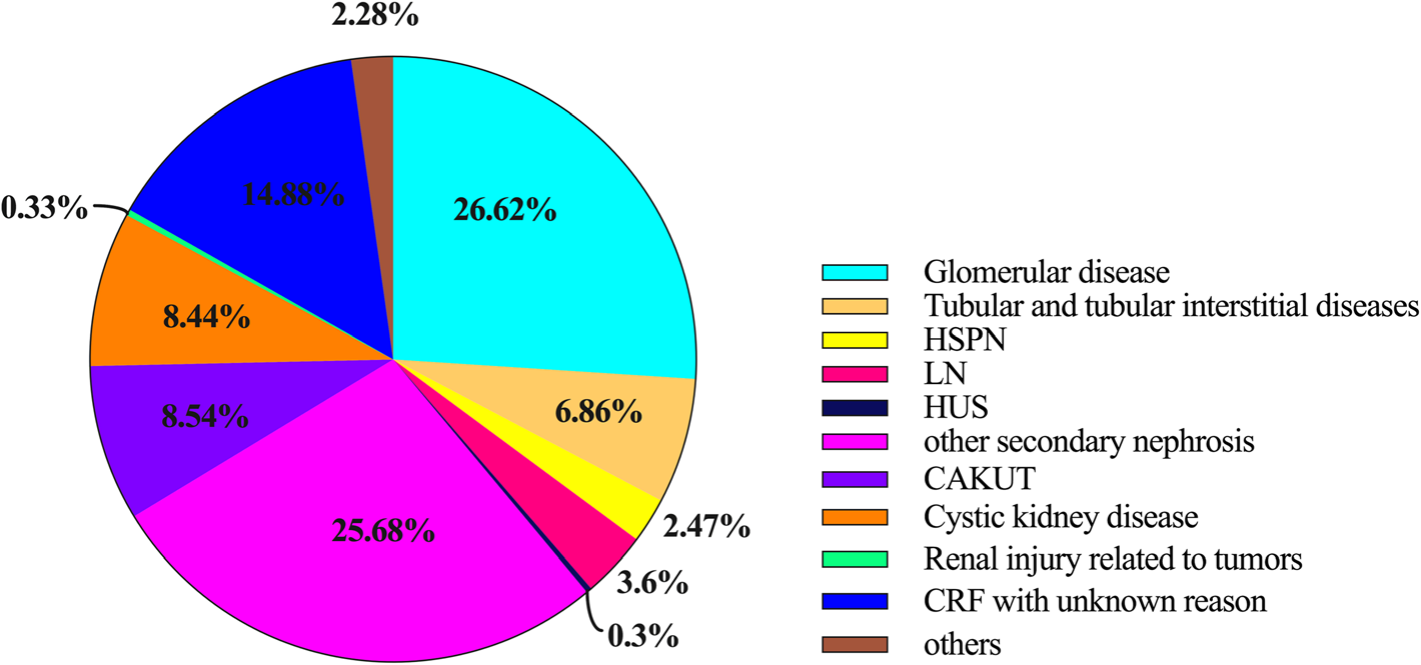
Etiology of CKD among CKD-label patients

Among CKD-label patients, secondary nephrosis was the etiology in 37.95 % of cases, of which the percentages of HSPN, LN, HUS and other secondary nephrosis were 2.47 %, 3.6 %, 0.3 and 25.68 %, respectively. CAKUT, glomerular diseases and cystic kidney disease accounted for 8.54 %, 26.62 and 8.44 %, respectively. Tubular disease and tubular interstitial diseases accounted for 6.86 % of cases (Fig. 7).

## Discussion

To our knowledge, this is the first large-scale study of childhood CKD in China, with data collected from the nationwide HQMS database. The HQMS database includes hospitals from throughout China. Moreover, all included institutions are tertiary hospitals with high-quality physicians who are capable and qualified to diagnose and treat CKD, which makes the database of high quality and the data of high security and reliability.

It was interesting that the number of male patients was 1.8 times that of female patients. It was surprising that only 2.37 % of patients had a discharge diagnosis of CKD (“CKD-label”), clearly indicating that many physicians had low diagnostic awareness of CKD. Nevertheless, physicians’ awareness gradually increased, from 2.61 % to 2013 to 3.09 % in 2016. As early as 2005, Stevens et al. reported a lack of awareness of CKD among physicians in the US [18], which they attributed to the lack of awareness of guidelines that define CKD irrespective of cause or discordance between serum creatinine and estimated GFR. Late detection of CKD can lead to delays in diagnosis, treatment and referral to nephrologists, which may result in adverse outcomes associated with CKD [19, 20]. Physicians’ awareness of CKD is of vital importance to achieve improved outcomes. Low rates of diagnosis and awareness of CKD in our study indicate physicians’ lack of awareness of CKD.

Over 35 % of pediatric CKD-label patients were in CKD stage 5, which suggests that doctors have greater diagnostic awareness of the most severe stage of renal disease. Similarly, a multicenter study in Turkey reported that almost one-third of patients were in stage 5, indicating that earlier stages of CKD were not recognized by physicians or that patients with low socioeconomic status lacked access to healthcare facilities [21]. The renal function of patients with CKD stage 5 is so impaired that doctors more easily recognize the disease. Inversely, patients with CKD stages 1 to 4 have less renal function impairment, and thus doctors have lower diagnostic awareness. However, CKD can progress to ESRD from all stages, a fact that needs recognition to slow down disease progression and decrease ESRD occurrence.

The percentage of pediatric CKD patients with CAKUT was 29.68 %, whereas glomerular diseases accounted for 21.18 % of cases; these findings were consistent with data from other reports. Many European countries, the United States, Australia and New Zealand have reported that congenital disorders and/or hereditary diseases are the most frequent primary renal cause of CKD and ESRD, accounting for 21.8–59 % of primary renal cases of ESRD, whereas glomerular diseases accounted for 11.9–32.5 % of cases [9, 22–25]. In other countries, glomerular diseases are the most common primary diseases causing CKD. A cross-sectional study in Iran [26] showed that glomerulonephropathy and congenital urological malformations were responsible for 35.2 and 28.2 %, respectively, of cases of pediatric CKD, stages 3 to 5. Another cross-sectional study in Brunei Darussalam [27] reported that glomerulonephritis accounted for 69 % and CAKUT for 20 % of cases of CKD in children and adolescents. A multicenter analysis of CRF in the pediatric population in China [10] showed that glomerular diseases were the leading cause of CKD, accounting for 70 % of cases, whereas congenital and hereditary diseases accounted for only 17.7 % of CRF cases. The current study also showed an increasing proportion of CAKUT cases and a decreasing proportion of glomerular diseases over the 4-year study period. Herein, we compared our research data with the reported results of CKiD [28] and 4 C study [29]. Notably, the percentage of glomerular disease in our study (21.18 %) was similar with reported percentage in CKiD study (21.72 %). However, in 4 C study, the reported percentage of glomerular disease was only 7.6 %, which were significantly lower. For CAKUT and tubular interstitial diseases, the percentage in our study (24.61 and 2.79 %, respectively) were also obviously lower than reported percentage (71.1 and 13.0 %) in 4 C study. The reported difference may be explained by the geographically epidemiological variance and genetic ethnicity variance between Chinese and Caucasian.

As for renal biopsy frequencies of CKD, IgA nephropathy were the leading diagnosis, which accounted for more than 50 %. Similar result was found in an analysis of 50 759 biopsy-proven adult cases in China [30] as well as in pediatric patients in Taiwan of China [31]. Differently, FSGS was the primary diagnosis of renal biopsies in the USA [32], Argentina[33] and Turkey [21].

Among CKD-label patients, the etiologies of CKD were different from those in the overall CKD population. In the CKD-label group, the proportions of patients with glomerular diseases and CRF of unknown cause were higher, whereas the proportion with CAKUT was lower than in the overall CKD population. These differences indicate that physicians had higher diagnostic awareness of CKD in patients with glomerular diseases or CRF than in those with CAKUT.

### Strengths

First, so far as we know, this study is the first national survey of CKD in a pediatric population in China and includes the largest reported study population, which has a great impact on national policies on dialysis and organ allocations in children. Secondly, the process was rigorous, from study design to data capture and analysis. Data were captured from the database by using ICD-10 codes and were relatively accurate. Last but not least, we were able to recognize over 91 % of patients hospitalized in different hospitals, even in different cities, by searching name, sex and date of birth concurrently.

### Limitations

Although this hospitalized population-based study describes fundamental data on CKD in a pediatric population, under-reporting of CKD patients was inevitable for three reasons. First, the HQMS database includes only hospitalized patients. Secondly, although all tertiary hospitals in China are required to submit data to the HQMS system, not all tertiary hospitals are included in the database. Thirdly, because some primary diseases that lack ICD-10 codes were excluded from this study, some patients may not have been included in the analysis. There are intrinsic limitations in hospital discharge data that might be subject to a type of selection bias and the results cannot be the basis for population based estimates. Nevertheless, it does provide some basic information regarding the prevalence of these conditions among those who are hospitalized.

## Conclusions

This study provides a descriptive analysis of hospitalized pediatric CKD patients. Our study provides important and fundamental data for policy making and legislation, registry implementation, and the diagnosis, treatment and prevention of CKD in China. We propose to obtain improved data by using data of registry database towards understanding pediatric CKD in China in the concluding comments as the next steps.

## Supplementary Information

**Additional file 1. **The three editions of ICD-10 codes for renal disease of CKD.

## Data Availability

All data are included in the manuscript and supplementary materials.

## Abbreviations

CAKUT: Congenital anomalies of kidney and urinary tract
CKD: Chronic kidney diseases
CRF: Chronic renal failure
ESRD: End-stage renal diseases
HQMS: Hospital Quality Monitoring System
HSPN: Henoch-Schonlein purpura nephritis
HUS: Hemolytic uremic syndrome
ICD: International Classification of Diseases
LN: Lupus nephritis
